# TlyC, a conserved hemolysin in *Rickettsia*, contributes to spotted fever pathogenesis in mice

**DOI:** 10.1128/spectrum.00303-25

**Published:** 2025-08-12

**Authors:** Luke Helminiak, Smruti Mishra, Ivy Lu, Hwan Keun Kim

**Affiliations:** 1 Center for Infectious Diseases, Stony Brook University 12301, Stony Brook, New York, USA; 2 Department of Microbiology and Immunology, Stony Brook University 273107, Stony Brook, New York, USA; University of Nebraska Medical Center, Omaha, Nebraska, USA

**Keywords:** *Rickettsia*, hemolysis, TlyC, secretion, pathogenesis

## Abstract

*Rickettsia* circulates between mammalian hosts and hematophagous arthropod vectors by exploiting their intracellular environment. With advances in rickettsial genetic tools, recent studies have identified novel molecular mechanisms involved in *Rickettsia*-host-vector interactions. However, a significant knowledge gap exists in understanding how *Rickettsia* modulates its virulence functions to survive in two drastically different environments (mammalian hosts vs arthropod vectors). Bacterial hemolysins play a crucial role in neutralizing innate immune functions through pore-forming activities and direct interactions with cognate receptors. Prior work suggested that typhus group *Rickettsia* (e.g., *R. typhi* and *R. prowazekii*), but not spotted fever group *Rickettsia* (e.g., *R. conorii* and *R. parkeri*), can induce hemolytic activities upon direct interactions with red blood cells. Here, we demonstrate that typhus and spotted fever groups exhibit comparable hemolytic activities. Furthermore, by characterizing an *R. conorii* transposon insertional variant (HK27), we document that TlyC, a factor conserved in *Rickettsia*, is responsible for pH-, temperature-, and host species-dependent hemolytic activities. Our biochemical and genetic studies confirmed that the first 10 amino acids are critical in facilitating hemolytic activities without affecting TlyC localization to the outer membrane. Compared to wild-type *R. conorii*, the HK27 variant showed reduced intracellular survival in primary endothelial cells and attenuated virulence in mice. These findings suggest a functional role for a conserved hemolysin in rickettsial pathogenesis.

**IMPORTANCE:**

Rickettsiosis is a vector-borne disease that causes systemic and potentially fatal vasculitis if not diagnosed promptly and treated with antibiotics. Pathogenic *Rickettsia* species, such as *Rickettsia conorii*, preferentially infect vascular endothelial cells with extensive abilities to survive in the cytoplasm of professional phagocytes. With the development of genetic tools for *Rickettsia*, recent studies have highlighted the biological roles of unique and conserved factors involved in rickettsial pathogenesis and vector transmission. However, additional studies are warranted to uncover essential molecular mechanisms that can be exploited to generate vaccines or therapeutics. The significance of our research is the identification of a conserved hemolysin exhibiting unconventional hemolytic activities and its contribution to rickettsial pathogenesis. Our research establishes a concrete foundation for studying protein secretion pathways that translocate effector proteins in *Rickettsia*, understanding how *Rickettsia* controls host cell membrane disruption, and identifying factors that support the rickettsial lifecycle between arthropod vectors and mammalian hosts.

## INTRODUCTION

*Rickettsia* is a genus of gram-negative α-proteobacterial organisms exhibiting unique obligate intracellular lifecycles in hematophagous arthropods (e.g., ticks, fleas, or lice) and mammalian hosts (1). Based on genetic and biological attributes, rickettsiae are categorized into four different groups: spotted fever group (SFG), typhus group (TG), transitional group (TRG), and ancestral group (AG) (2). Through its reductive genome evolution, rickettsiae have shed unnecessary genetic information while retaining genes important for survival within arthropod vectors, vertical and horizontal transmission, and pathogenesis in mammalian hosts (3, 4). Despite the technical difficulties in working with obligate intracellular parasitic organisms, recent studies have developed genetic tools and characterized the underlying molecular mechanisms involved in rickettsial intracellular survival, immune evasion, vector transmission, and pathogenesis (5–9). However, further studies are necessary to uncover the novel biological functions of conserved factors for rickettsial transmission and pathogenesis.

In 1948, Clarke and Fox reported a manuscript establishing the fundamental basis of *in vitro* hemolytic activities of TG rickettsiae (10). In their work, the authors reported that freshly prepared TG rickettsiae exhibited host- (hemolytic activities with red blood cells [RBCs] prepared from sheep and rabbit, but not from mice, cotton rats, and guinea pigs) and temperature- (hemolytic activities at 35°C, but not at 4°C) dependent hemolytic activities. Rickettsial hemolysis progressed slowly (over 24 hours) and never reached a complete lysis. The hemolytic factor was inseparable from rickettsial bodies and neutralized by hyperimmune serum against whole-cell rickettsiae. Interestingly, *Orientia tsutsugamushi*, a causative agent of scrub typhus, did not exhibit hemolytic activities under the same experimental conditions. In subsequent studies, investigators confirmed rickettsial hemolytic activities using *in vitro* and *in vivo* approaches and suggested that TG rickettsiae induce pH- and contact-dependent hemolysis (11–14). Notably, one study documented that cholesterol facilitates rickettsial adsorption and hemolysis, implicating that TG *Rickettsia* may produce cholesterol-dependent cytolysins (CDC) (15).

Bacterial pore-forming toxins (PFT) exhibit diverse virulence functions, assisting in immune evasion and pathogenesis (16). Over the last decades, numerous studies have documented the biochemical and pathophysiological attributes of PFTs, such as perfringolysin of *Clostridium perfringens*, pneumolysin of *Streptococcus pneumoniae*, streptolysin O of *Streptococcus pyogenes*, listeriolysin O of *Listeria monocytogenes*, and α-hemolysins of *Escherichia coli* and *Staphylococcus aureus*. Prior studies documented putative functional roles of a rickettsial hemolysin (TlyC) by expressing *Rickettsia prowazekii tlyC* or *Rickettsia typhi tlyC* in heterologous bacterial organisms, such as *Salmonella enterica* serovar Typhimurium, *E. coli*, and *Proteus mirabilis* (17, 18). The expression of *R. typhi* TlyC in *E. coli* or *P. mirabilis* conferred *in vitro* hemolytic activities when incubated with sheep RBCs (17). On the other hand, *S. enterica* expressing *R. prowazekii* TlyC escaped the *Salmonella*-containing vacuoles and survived within the cytoplasmic compartment of Vero cells (18). These studies suggest that TlyC may contribute to rickettsial pathogenesis. However, the specific mechanisms by which TlyC facilitates membrane disruption remain unclear.

In the present study, we utilized a transposon insertional variant of *Rickettsia conorii* to identify TlyC as a major rickettsial hemolysin. We further demonstrate that TlyC is a conserved determinant exhibiting pH-, temperature-, and host species-dependent hemolytic activities. Lastly, our present work suggests that TlyC is deposited on the outer membrane of rickettsiae through an undetermined secretion pathway and contributes to rickettsial intracellular survival and pathogenesis.

## MATERIALS AND METHODS

### Cell lines and bacterial strains

Vero cells (African green monkey kidney cells, ATCC) were cultured in Dulbecco’s modified Eagle’s medium (DMEM, Sigma) supplemented with 10% heat-inactivated fetal bovine serum (HI-FBS, Gibco) at 37°C in a 5% CO_2_ atmosphere. Human dermal microvascular endothelial cells (HDMEC, PromoCell) were grown in endothelial cell basal medium (C-22221, PromoCell) mixed with Supplement Pack (C-39221, PromoCell) at 37°C in a 5% CO_2_ atmosphere. *E. coli* DH5α, BL21, and XL-1 were grown in Luria-Bertani Miller broth (LB, Difco) at 37°C, supplemented with ampicillin (100 µg·mL^−1^), rifampin (20 µg·mL^−1^), or chloramphenicol (20 µg·mL^−1^). Stocks of *R. conorii* strain Malish 7 (VR613, ATCC), *Rickettsia parkeri* strain Maculatum C (NR-10402, BEI Resources), and *R. typhi* strain Wilmington (a kind gift from Dr. Hackstadt, Rocky Mountain Laboratories, National Institutes of Health [NIH]) were generated by growing them in Vero cells at 34°C in a 5% CO_2_ atmosphere and subjecting to differential centrifugation through 25% MD-76R solution (816 mM meglumine diatrizoate, 157 mM sodium diatrizoate hydrate, 1 mM NaH_2_PO_4_, pH 7.0; 21,000 × *g*, 4°C, and 45 minutes). Rickettsial samples were stored at −80°C in sucrose phosphate glutamate (SPG) buffer (218 mM sucrose, 3.8 mM KH_2_PO_4_, 7.2 mM K_2_HPO_4_, and 4.9 mM L-glutamate, pH 7.4). *R. conorii* variants were grown in Vero cells in the presence of rifampin (0.2 µg·mL^−1^) and/or chloramphenicol (0.3 µg·mL^−1^). Rickettsial genomic DNA samples were Illumina sequenced on a NextSeq 550 instrument to confirm their sequence identities (PureLink Genomic DNA Mini Kit, Invitrogen, NCBI Reference Sequences: NC_003103.1, NC_017044.1, and NC_006142.1).

### Plasmid construction

Plasmids were constructed using the primers and restriction enzymes indicated in Table S1. For the generation of complementing plasmids, recombinant gene fragments were synthesized for the endogenous promoter region (255 base pairs upstream of the start codon), followed by *R. conorii tlyC* (*tlyC*_1–299_, *tlyC*_Δ2–11_, or *tlyC*_Δ2–21_) with endogenous terminator (100 base pairs downstream of the stop codon, synthesized by Twist Bioscience). PCR-amplified gene fragments were cloned into the multiple cloning sites of pHTRL7, resulting in the generation of pTlyC, pTlyC_Δ2–11_, and pTlyC_Δ2–21_, respectively. To generate N-terminal His_6_-tagged recombinant TlyC_Δ2–51_, a recombinant gene fragment was synthesized (Integrated DNA Technologies) and PCR-cloned into a pET15b expression vector. All plasmid preparations were sequence verified.

### Rabbit immunization

pET15b-TlyC_Δ2-51_ plasmid was transformed into *E. coli* BL21(DE3). Overnight cultures of transformants were diluted 1:100 into fresh LB media and grown at 37°C to an OD_600_ of 0.5, at which point cultures were induced with 1 mM isopropyl β-D-1-thiogalatopyranoside and grown for an additional 3 hours. Bacterial cells were sedimented by centrifugation, suspended in column buffer (50 mM Tris–HCl pH 7.5, 150 mM NaCl), and disrupted with a French pressure cell. Lysates were cleared of membrane and insoluble components by ultracentrifugation. Proteins in the soluble lysate were subjected to nickel-nitrilotriacetic acid affinity purification. Proteins were eluted in column buffer with 500 mM imidazole, buffer-exchanged using HiTrap desalting column (GE Healthcare) equilibrated with 50 mM phosphate-buffered saline, pH 7.4, and subjected to size-exclusion chromatography (AKTA FPLC). Eluted samples were analyzed using Coomassie-stained SDS-PAGE. Fractions containing recombinant TlyC_Δ2–51_ were subjected to bicinchoninic acid assay (Thermo Scientific). For antibody generation, rabbits (Charles River Laboratories, 6-month-old New Zealand white female) were immunized with 200 µg protein emulsified in Complete Freund’s Adjuvant (Difco) via subscapular injection. For booster immunizations, proteins were emulsified in incomplete Freund’s adjuvant and injected 21 or 42 days following the initial immunization. On day 60, rabbits were terminally bled, and serum was recovered.

### Transformation

Approximately 5 × 10^7^ PFU of Renografin-purified *R. conorii* variants were electroporated with 5–10 µg of plasmids and immediately plated onto Vero cell monolayers. After 60 minutes of infection at 34°C, the medium was removed and replaced with an agarose overlay containing DMEM 5% HI-FBS and 0.5% agarose. Six hours post-infection, growth media with antibiotics were added to select transformants. Individual plaques were isolated and expanded in Vero cell monolayers in the presence of antibiotics.

### Growth and plaque assay

Growth curves were generated by infecting monolayers of HDMECs with *R. conorii* wild-type (WT) or variants in six-well plates at a multiplicity of infection (MOI) of 0.01 without antibiotics. At 2-day intervals, HDMECs in each well were dislodged with sterile glass beads and lysed by vortexing with glass beads. Infectious titers were determined by infecting monolayers of Vero cells with 10-fold serial dilutions of lysates containing *Rickettsia* in DMEM supplemented with 5% HI-FBS. Upon infection, Vero cells were incubated at 34°C with 5% CO_2_ for 60 minutes to allow attachment and overlaid with DMEM containing 5% HI-FBS and 0.5% agarose. Plaque numbers were determined on day 5 or 6 post-infection. Differential interference contrast images of HDMEC cells infected with *Rickettsia* were acquired with Eclipse TE300 Inverted Microscope (Nikon) using NIS-Elements Basic Research software (Nikon). All images were contrast-adjusted and analyzed using ImageJ (NIH). To determine the genetic stability, individual plaques were isolated from Vero cells (infected with HDMEC day 6 post-infection lysates) on day 5 post-infection and expanded for 5 days in Vero cells. Vero cells were dislodged with sterile glass beads and passed through a blunt-end 28-gauge needle. Lysates were cleared of large cellular debris by centrifugation (1,000 × *g*, 5 minutes). After another round of centrifugation to wash and pellet rickettsiae (17,000 × *g*, 15 minutes), genomic and plasmid DNA were extracted (PureLink Genomic DNA Mini kit, Invitrogen) to confirm the presence of *kkaebi* transposon by PCR (primers: Tn5F/R and GenomicTlyCF/R, Table S1) and pTlyC (primers: pHTRL7MCSF/R, Table S1).

### Hemolysis assay

Sheep defibrinated blood (Hemostat Labs) or RBCs derived from human, cow, rabbit, dog, sheep, and guinea pig (Innovative Research) in 300 µL SPG was incubated with 200 µL of 4 × 10^6^ PFU *Rickettsia*, SPG (negative control), or MQ H_2_O (positive control) at 24°C, 34°C, or 37°C. To adjust pH, sheep RBCs were gently washed in 50 mM HBSS (pH 7.0) or 50 mM sodium acetate (pH 5.0). Spectrophotometric analyses (*A*545) were performed to determine hemoglobin release in individual samples at 1, 12, and 24 hours post-incubation. Percent hemolysis was calculated by normalizing values relative to positive controls at each time point. Mock samples lacking rickettsiae were used to measure spontaneous hemolysis under the same experimental conditions. In addition, plaque assays were performed to determine rickettsial survival at 1, 12, and 24 hours post-incubation and to measure rickettsial adsorption to sheep RBCs at 1 and 12 hours post-incubation. For rickettsial adsorption analysis, samples were centrifuged (1,000 × *g*, 5 minutes) to separate the supernatant (non-adsorbed rickettsiae) from the pellet (adsorbed rickettsiae). The pellets were washed twice in 100 µL SPG before the plaque assay. The rickettsial abundance was normalized to the total rickettsial abundance at each time point. Mock refers to *R. conorii* WT only control samples (without sheep RBCs).

### Membrane fractionation

Membrane fractionation was conducted based on a previously published protocol (19, 20). The centrifugation sediments of *Rickettsia* (approximately 6.25 × 10^7^ PFU) were washed in 1 mL of 20 mM Tris pH 8.0 and resuspended in 180 µL of 20 mM Tris pH 8.0 supplemented with 20% sucrose. Next, samples were incubated with lysozyme (0.5 mg·mL^−1^) for 40 minutes on ice, followed by DNase I (four units) treatment for 20 minutes on ice. Samples were centrifuged at 17,000 × *g* for 20 minutes at 4°C. The supernatant was treated with lysozyme (0.1 mg·mL^−1^) for 60 minutes on ice (Periplasm). The pellet was resuspended in 180 µL of 20 mM Tris pH 8.0 supplemented with a protease inhibitor cocktail (Roche). Samples were subjected to eight rounds of sonication on ice (Qsonica Q125, power 5, amplitude 40%, 15 seconds on, 45 seconds off), then centrifuged at 17,000 × *g* for 15 minutes at 4°C to remove intact rickettsial cells. The supernatant was ultracentrifuged at 115,000 × *g* for 70 minutes at 4°C. After centrifugation, the supernatant was separated (Cytosol). The pellet was washed in 200 µL of 20 mM Tris pH 8.0 and resuspended in 180 µL of 20 mM Tris pH 8.0 supplemented with 2% Triton X-100 and protease inhibitor cocktail (Roche). The mixture was ultracentrifuged at 115,000 × *g* for 70 minutes at 4°C, separating the supernatant (Inner membrane) from the pellet (Outer membrane). The pellet was resuspended in 180 µL of 200 mM Tris pH 8.8 supplemented with 2% SDS and 10 mM EDTA and incubated for 16 hours at 4°C.

### Whole-cell lysate preparation

The centrifugation sediments of *Rickettsia* (approximately 6.25 × 10^7^ PFU) were washed in 1 mL of 20 mM Tris pH 8.0 and resuspended in 153.8 µL of 20 mM Tris pH 8.0 supplemented with a protease inhibitor cocktail (Roche). Samples were subjected to eight rounds of sonication on ice (Qsonica Q125, power 5, amplitude 40%, 15 seconds on, and 45 seconds off), then incubated with lysozyme (0.5 mg·mL^−1^) and DNase I (four units) for 60 minutes on ice. Then, the samples were mixed with 20 µL of 200 mM Tris pH 8.8 supplemented with 20% SDS and incubated for 16 hours at 4°C.

### SDS-PAGE and immunoblotting

Samples were mixed with sample buffer (125 mM Tris-HCl, 4% SDS, 20% glycerol, 10% 2-mercaptoethanol, and 0.01% bromophenol blue, pH 6.8) and boiled at 95°C for 10 minutes. Samples were separated on 15% SDS-PAGE gels and stained with Coomassie Brilliant Blue R-250 to assess the total protein abundance. For immunoblot analyses, samples were electrophoretically transferred from the gel onto a 0.22 µm polyvinylidene difluoride membrane (Millipore Sigma). The membrane was immersed in blocking buffer (Tris-buffered saline [TBS; 100 mM Tris-HCl and 150 mM NaCl, pH 7.6] with 5% milk) for 90 minutes at room temperature. The membrane was washed and incubated in TBS containing primary antibodies or antisera for 18 hours at 4°C. Each membrane was used for a single primary antibody or antiserum to avoid cross-reactive or overlapping immunoreactive signals. Rabbit antisera specific to TlyC_Δ2–51_ was used at a 1:5,000 or 1:10,000 dilution. Mouse monoclonal antibody detecting RpoA was used at a 1:5,000 dilution (clone 4RA2, BioLegend). Recombinant mouse monoclonal antibody detecting OmpA (clone 13-3) was used at a 1:10,000 dilution (GenScript). The membrane was washed three times and incubated with peroxidase-conjugated secondary antibodies (anti-mouse IgG and anti-rabbit IgG [Rockland]) or IRDye 680RD-conjugated secondary antibodies (anti-mouse IgG and anti-rabbit IgG [Li-Cor]) at a 1:10,000 dilution in TBS for 2 hours at room temperature. After a final wash, the membrane was developed using Amersham Hyperfilm ECL (GE Healthcare), ImageQuant LAS500 (GE Healthcare), or Odyssey CLx (Li-Cor). Signal intensities were calculated using ImageJ (NIH) or Image Studio (Li-Cor) and normalized to internal controls.

### Mouse model of spotted fever

C3H/HeN mice (6 weeks, Charles River Laboratories) were infected by intravenous retro-orbital injection with 1 × 10^3^ PFU of *R. conorii* variants in 0.1 mL SPG buffer. Infected mice were monitored twice daily for signs of disease and daily for weight loss. Blood samples were collected on day 14 post-infection to determine TlyC-specific antibodies by western blot analyses.

### Biosafety and biosecurity

This study was carried out in strict accordance with the recommendations in the Guide for the Care and Use of Laboratory Animals of the NIH. Research was performed in accordance with institutional guidelines following experimental protocol review, approval, and supervision by the Institutional Biosafety Committee (IBC, 1445506) and Institutional Animal Care and Use Committee (IACUC, 1456687) at Stony Brook University. The IBC and the NIH Recombinant DNA Advisory Committee reviewed and approved the use of a gene encoding chloramphenicol resistance for selecting mutants with insertional *kkaebi* lesions in *R. conorii*. The Division of Animal Laboratory Research at Stony Brook University operates in accordance with the American Association for Laboratory Animal Science, the American College of Laboratory Animal Medicine, and Animal Welfare Assurance ID D16-00006 (A3011-01) of the NIH. Rabbits were individually identified by ear tattoo and housed in standard cages with the room temperature maintained at 20 to 22°C and relative humidity between 30% and 70%. Rabbits had *ad libitum* access to water, timothy hay, and a pelleted commercial diet, as well as fresh fruit and vegetable enrichment. Experiments involving infectious *Rickettsia* were performed in the animal biosafety level 3 containment facility. Mice were housed in either standard filter-topped shoebox micro-isolator cages or filter-ventilated cages. Rooms had 10–15 air changes per hour and were maintained at 70°F–72°F. Pelleted irradiated Purina mouse chow was provided *ad libitum*, and hyper-filtered water (2 µm) was provided via water bottles *ad libitum*. All mice were provided with Enviro-Dri nesting material. Mice were euthanized with 3 L/minute carbon dioxide inhalation, consistent with the recommendations of the panel on euthanasia of the American Veterinary Medical Association and Stony Brook University IACUC.

### Statistical analyses

All statistical analyses were performed using Prism software (GraphPad, version 10.4.2). Two-/one-way analysis of variance (ANOVA) with Tukey’s multiple comparison test was performed to determine the statistical significance of hemolysis data. Two-way ANOVA with Sidak’s multiple comparison test determined the statistical significance of rickettsial replication and mouse body weight changes. One-way ANOVA with Tukey’s multiple comparison test was used to analyze the statistical significance of TlyC secretion data. One-way ANOVA with Dunnett’s multiple comparison test was performed to determine the statistical significance of relative plaque sizes. Lastly, a log-rank test was performed to determine the statistical significance of mouse mortality data.

## RESULTS

### Spotted fever rickettsiae exhibit hemolytic activities

Recent whole-genome sequencing and bioinformatic analyses identified three putative hemolysins in *Rickettsia: tlyC* (*Rc1141*), *tlyC2* (*Rc1079*), and *tlyA* (*Rc0822*). All three genes are highly conserved and unique in *Rickettsia*, with lower homologies in AG rickettsiae (Table 1). In previous studies, investigators observed the presence of *tlyC*-specific PCR amplification in TG rickettsiae but not in other groups (17). Thus, it was presumed that TlyC-dependent hemolytic activity is a unique feature of TG rickettsiae. However, our bioinformatic analysis identified TlyC with varying amino acid sequence homologies in all groups. Interestingly, TlyC contains two tandem repeats of the cystathionine β-synthase domains (TlyC_75–193_), highly conserved in proteins of unknown functions or putative hemolysins, and a CorC_HlyC domain (TlyC_213–292_) whose function is unclear but predicted to be involved in ion transportation.

**TABLE 1:** Amino acid sequence identity of putative hemolysins in *Rickettsia*

Groups^*a*^	Species^*b*^	TlyA^*c*^		TlyC2^*c*^		TlyC^*c*^		
		% Identity^*d*^	Length^*e*^	% Identity^*d*^	Length^*e*^	% Identity^*d*^	Length^*e*^	N-terminal A.A.^*f*^
SFG	*R. conorii*	100.00	251	100.00	424	100.00	299	MLKSSKKEDSS
	*Rickettsia africae*	99.20	251	99.29	424	99.33	299	MLKSSKKEDSS
	*Rickettsia peacockii*	99.80	253	99.06	424	99.33	299	MLKSSKKEDSS
	*Rickettsia sibirica*	99.80	251	99.76	424	98.66	299	MLKSSKKEDSS
	*R. parkeri*	99.20	251	99.53	424	98.66	299	MLKSSKKEDSS
	*Rickettsia montanensis*	97.61	251	98.35	424	98.33	299	MLKSSKKEDSS
	*Rickettsia amblyommatis*	96.02	251	98.35	424	97.99	299	MLKSSNKEDSS
	*Rickettsia helvetica*	92.68	247	98.82	424	96.64	301	MLKSSKKEDSS
	*Rickettsia australis*	91.87	247	97.41	424	96.64	301	MLKSSKKEDSS
TG	*R. prowazekii*	86.06	251	94.61	424	91.64	303	MLKSSKHEDSS
	*R. typhi*	86.45	251	94.61	424	90.97	303	MLKSSKHEDSS
TRG	*Rickettsia felis*	94.02	254	98.35	424	97.99	299	MLKSSKKEDSS
	*Rickettsia akari*	91.06	247	95.99	424	95.97	301	MLKSSKKEDSS
AG	*Rickettsia canadensis*	89.84	256	96.46	424	91.23	298	MFKSSKKEDSS
	*Rickettsia bellii*	79.67	249	86.56	424	82.43	301	MLKSSKKEDDS

^*a*^ Different groups of rickettsial organisms: SFG, TG, TRG, and AG.

^*b*^ Rickettsial species belonging to four different groups.

^*c*^ Three gene products annotated as hemolysins in *Rickettsia*.

^*d*^ Percent amino acid sequence identity compared to *R. conorii*.

^*e*^ Lengths of individual polypeptides.

^*f*^ The first 10 amino acids of TlyC.

**Fig 1:**
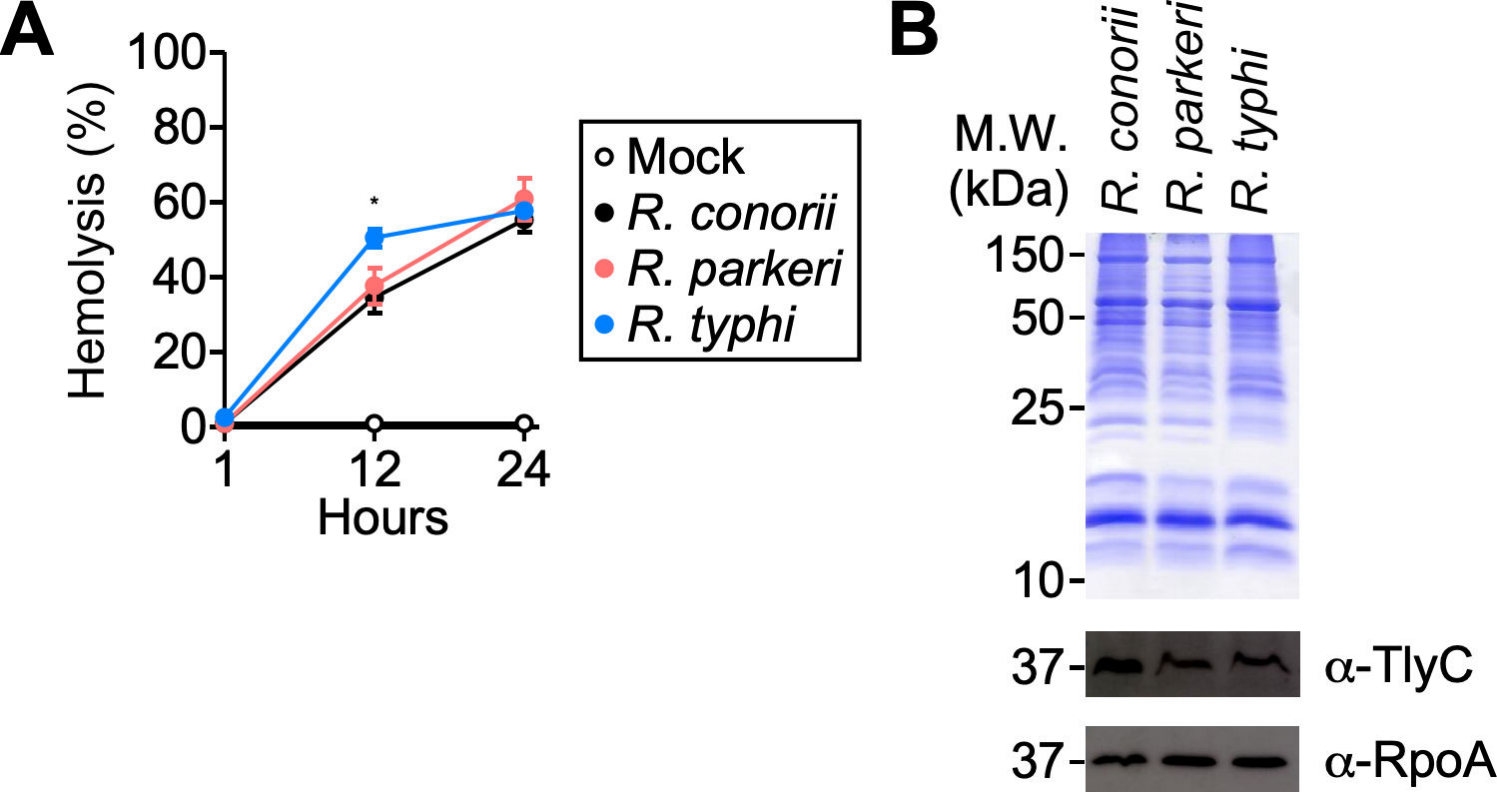
SFG and TG rickettsiae exhibit hemolytic activities. (**A**) Hemoglobin release was measured in samples incubated with 4 × 10^6^ PFU *Rickettsia* with defibrinated sheep erythrocytes at 37°C (*N* = 3, mean ± SD). Two-way ANOVA with Tukey’s multiple comparisons test was performed, *P < 0.05. (**B**) Representative Coomassie-stain and western blot analyses of whole-cell lysate samples (*N* = 3) prepared from *R. conorii*, *R. parkeri*, and *R. typhi*.

Since *tlyC* is conserved in *Rickettsia*, we hypothesized that rickettsiae belonging to the SFG and TG exhibit comparable hemolytic activities. To test this, we incubated Renografin-purified *R. conorii* (SFG), *R. parkeri* (SFG), and *R. typhi* (TG) with defibrinated sheep RBCs at 37°C. At 1, 12, and 24 hours post-incubation, we measured the optical density (545 nm) to detect the released hemoglobin (Fig. 1A). As reported in prior studies, *R. typhi* caused slow and incomplete lysis of sheep RBCs. Interestingly, we observed comparable hemoglobin levels in samples incubated with *R. conorii* and *R. parkeri*, suggesting that SFG rickettsiae also exhibit hemolytic activity under this experimental condition. Of note, all samples exhibited minimal levels of spontaneous hemolysis, indicating that hemoglobin is released as a direct consequence of rickettsial incubation. Lastly, using TlyC_Δ2-51_-specific rabbit polyclonal antibodies, we detected TlyC in whole-cell lysates of *R. conorii*, *R. parkeri*, and *R. typhi* (Fig. 1B). Our results demonstrate that SFG and TG rickettsiae exhibit comparable hemolytic activities and produce a conserved hemolysin, TlyC.

### TlyC is responsible for pH-, temperature-, and host-dependent hemolytic activities

**Fig 2:**
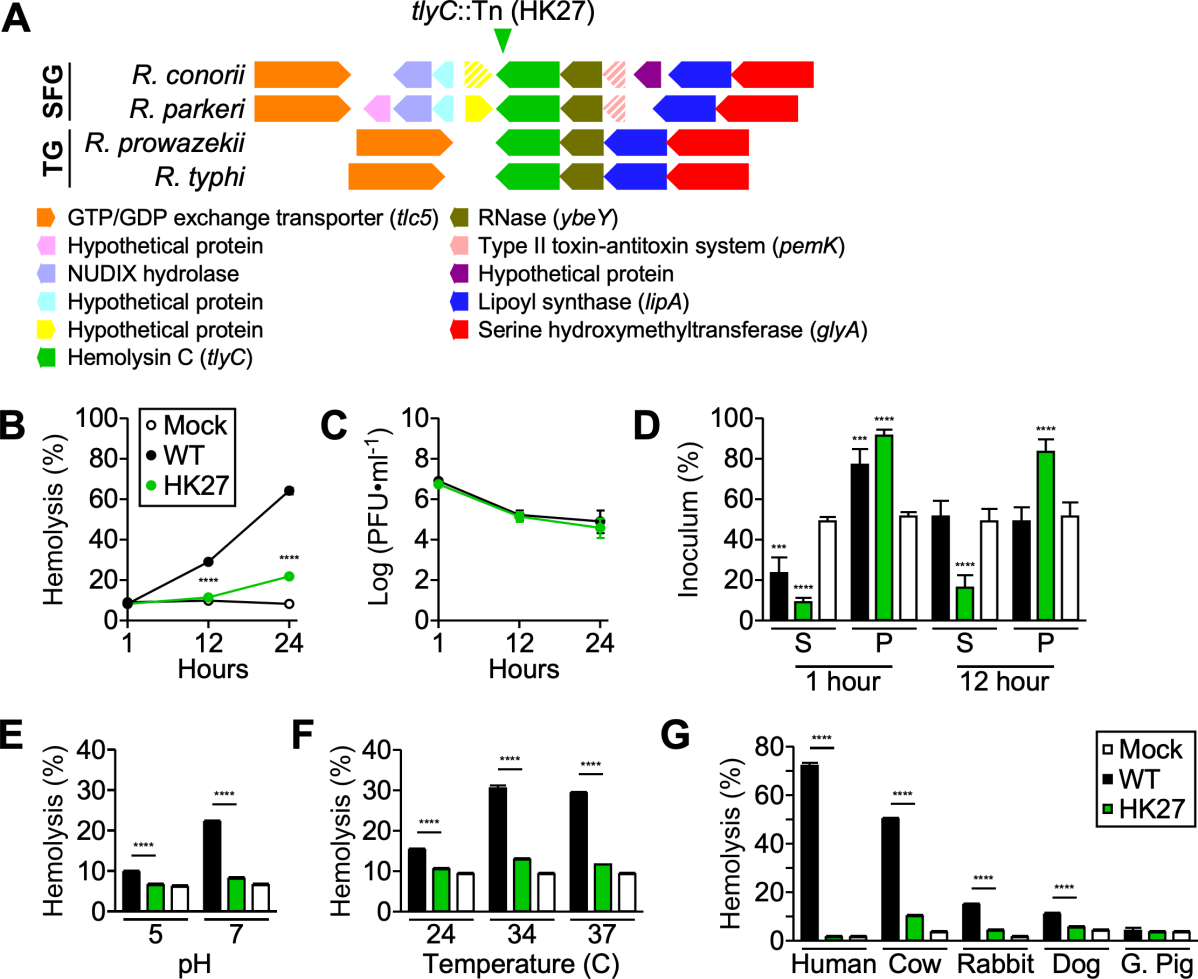
TlyC contributes to rickettsial hemolysis. (**A**) Diagram of genes surrounding *tlyC* in SFG and TG of *Rickettsia*. The arrowhead indicates a *kkaebi* transposon insertion site. Dashed lines indicate putative pseudogenes. (**B**) Hemoglobin release and (**C**) infectious titers were measured in samples (*N* = 3, mean ± SD) incubated with 4 × 10^6^ PFU *Rickettsia* with defibrinated sheep erythrocytes at 37°C at three different time points (1, 12, and 24 hours post-incubation). (**D**) Infectious titers were measured to determine *Rickettsia* adsorbed to sheep erythrocytes (pellet, P) or present in supernatant (S, *N* = 3, mean ± SD). Additional hemolysis assays (*N* = 3, mean ± SD) were performed (**E**) at two different pH (5 or 7, 34°C, 12 hours post-incubation), (**F**) at three different temperatures (24°C, 34°C, or 37°C, 12 hours post-incubation), or (**G**) with erythrocytes from humans, cows, rabbits, dogs, and guinea pigs (34°C, 12 hours post-incubation). Two-way ANOVA with Tukey’s multiple comparisons was performed; ***P < 0.001 and ****P < 0.0001.

**Fig 3:**
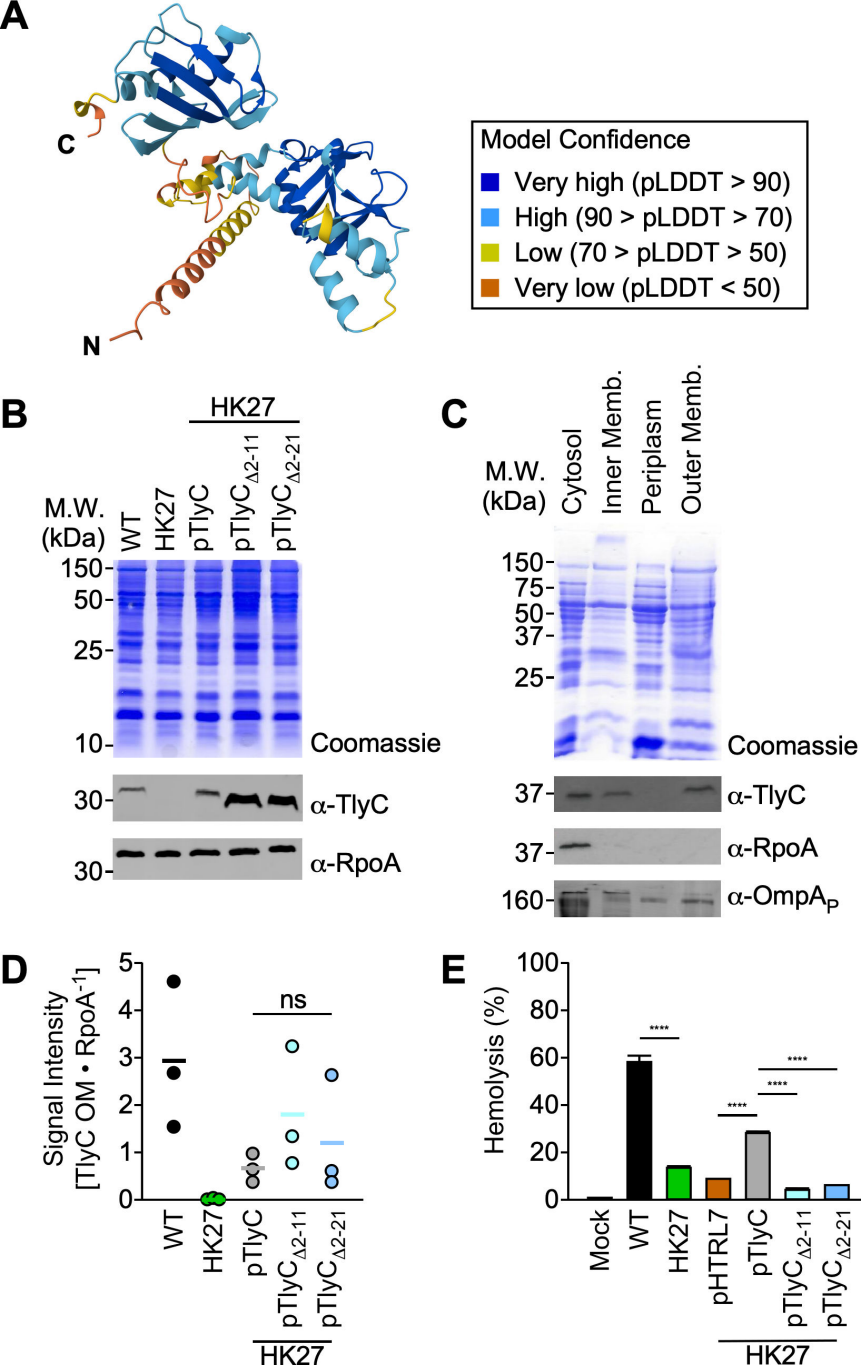
The first 10 amino acids of TlyC are dispensable for membrane translocation but essential for hemolysis. (**A**) AlphaFold prediction of TlyC structure. (**B**) Representative Coomassie stain and western blot analyses of whole-cell lysates (*N* = 3) prepared with *R. conorii* WT and HK27 expressing full-length TlyC or TlyC variants lacking the first 10 or 20 amino acids. (**C**) Representative Coomassie stain and western blot analyses of membrane fractionation samples (*N* = 3) prepared with *R. conorii* WT. (**D**) Normalized abundance of TlyC in the outer membranes of *R. conorii* WT and HK27 variants (*N* = 3). (**E**) Hemolytic activities were measured with *R. conorii* WT and HK27 variants (defibrinated sheep RBCs, 37°C, 12 hours post-incubation, *N* = 3, mean ± SD). One-way ANOVA with Tukey’s multiple comparisons test was performed, ****P < 0.0001, ns: not significant.

To determine whether TlyC contributes to rickettsial hemolytic activities, we utilized a transposon insertional variant (*tlyC*::Tn, HK27) harboring a *kkaebi* insertion in *Rc1141* (Fig. 2A). By western blot analysis, we confirmed that HK27 does not synthesize TlyC polypeptides (Fig. 3B). Compared to *R. conorii* WT, HK27 failed to lyse sheep RBCs at 12 hours post-incubation (Fig. 2B). Interestingly, HK27 displayed moderate hemolytic activity at 24 hours post-incubation, implicating that other rickettsial factors, such as putative hemolysins or phospholipases, may contribute to erythrocyte lysis. Of note, while the infectious titers for *R. conorii* WT and HK27 rapidly decreased in the first 12 hours of incubation, they remained comparable throughout the experiment, confirming that the inefficient hemolysis is due to the inability to synthesize TlyC in HK27 (Fig. 2C). Lastly, we determined that *R. conorii* WT adhered to RBCs, resulting in co-sedimentation with sheep RBCs at 1 hour post-incubation. However, after 12 hours of incubation, *R. conorii* WT exhibited minimal levels of co-sedimentation with RBCs (Fig. 2D). In contrast, HK27 remained adherent to RBCs for 12 hours, suggesting that *R. conorii* utilizes TlyC-independent mechanisms to interact with RBCs and requires TlyC-dependent hemolytic activities to disrupt and disengage from RBCs.

In their natural lifecycle, rickettsiae circulate between mammalian hosts and arthropod vectors, experiencing diverse environmental conditions. Prior studies documented that rickettsiae exhibit temperature-dependent hemolytic activities as the bacterial organisms fail to adsorb to erythrocytes and induce hemolysis at cold temperatures (14). In a separate study, the investigators documented optimal hemolytic activities at neutral pH (11, 12). By utilizing our transposon variant, we demonstrated that *R. conorii* WT, but not HK27, releases a significant level of hemoglobin at a neutral pH but shows limited hemolytic activity at acidic pH (Fig. 2E). In addition, we confirmed that *R. conorii* WT exhibits minimal hemolytic activity at room temperature (24°C) but induces significant and comparable hemolytic activity at skin (34°C) and body (37°C) temperatures (Fig. 2F).

Clarke and Fox documented that RBCs derived from mice, cotton rats, and guinea pigs resisted *Rickettsia*-induced hemolysis, implicating that *Rickettsia* may exhibit host-dependent hemolytic activities (10). Here, we incubated *R. conorii* WT and HK27 with RBCs extracted from humans, cows, rabbits, dogs, guinea pigs, and mice (Fig. 2G). Interestingly, *R. conorii* preferentially disrupted human and cow erythrocytes, exhibited moderate hemolysis toward rabbit and dog RBCs, and failed to release hemoglobin from guinea pig RBCs. Despite our repeated attempts, we could not test mouse erythrocytes under the same experimental conditions, primarily due to high levels of spontaneous lysis. Our results corroborate the differential hemolytic activities of *Rickettsia* and suggest that TlyC-host ligand interactions may contribute to differences in hemolysis kinetics.

### TlyC is deposited on the outer membrane of *Rickettsia*

Prior work documented that rickettsial hemolytic activities were inseparable from rickettsial bodies and suggested contact-dependent hemolytic mechanisms, implicating that the factors must be translocated to the outer membrane of *Rickettsia* (14). Unfortunately, our bioinformatic analyses failed to identify putative secretion signals involved in the Sec, Tat, Type I (T1SS), and Type IV secretion pathways (T4SS). Furthermore, TlyC does not appear to have transmembrane domains and acylation sites for membrane interactions. AlphaFold prediction of the TlyC structure identified two β-sheets flanked by α-helical bundles (Fig. 3A). In the predicted TlyC structure, the N-terminal region formed α-helical structures with low confidence, prompting us to investigate whether the N-terminal sequence has an atypical secretion signal for TlyC translocation in *Rickettsia*.

To study the biological attributes of the N-terminal amino acids in translocating TlyC to the outer membrane, we complemented HK27 with plasmids expressing a full-length TlyC (pTlyC) or variants lacking the first 10 or 20 amino acids (pTlyC_Δ2-11_ or pTlyC_Δ2-21_, respectively). Probing for TlyC in whole-cell lysate, we identified comparable levels of TlyC in HK27 pTlyC and abundant expression of TlyC variants in HK27 pTlyC_Δ2-11_ or HK27 pTlyC_Δ2-21_ (Fig. 3B). Next, we separated proteins in the subcellular compartments of *Rickettsia*. By probing for TlyC, we identified the presence of TlyC in the cytosol, inner membrane, and outer membrane fractions in *R. conorii* WT (Fig. 3C). As controls, we determined that RpoA is present in the cytosol but absent in other fractions. On the other hand, the majority of OmpA passenger domain lacking the β-barrel domain was detected in the periplasmic and outer membrane fractions. By quantifying signal intensities, we found that TlyC in the outer membrane of HK27 pTlyC was lower than WT but comparable to HK27 pTlyC_Δ2-11_ and HK27 pTlyC_Δ2-21_, implicating that the first 20 amino acids are dispensable for TlyC translocation to the outer membrane of *Rickettsia* (Fig. 3D). Since T1SS and T4SS signals are often encoded in the C-terminal residues, we generated another HK27 variant transformed with a plasmid (pTlyC_Δ249-299_) encoding TlyC without the last 50 amino acids. Despite repeated attempts and sequence confirmation, we were unable to detect TlyC_Δ249-299_ in HK27 using TlyC_Δ2-51_-specific rabbit antibodies (data not shown). Lastly, we performed the hemolysis assay to determine the functional consequences of losing the first 10 or 20 amino acids of TlyC (Fig. 3E). Surprisingly, although HK27 expressing full-length TlyC (HK27 pTlyC) partially complemented WT hemolytic activities, variants expressing TlyC_Δ2-11_ and TlyC_Δ2-21_ failed to release hemoglobin from sheep RBCs. Our results suggest that the first 20 amino acids of TlyC are dispensable for outer membrane translocation but essential for hemolytic activities.

### TlyC contributes to the intracellular replication of *Rickettsia* in endothelial cells

**Fig 4:**
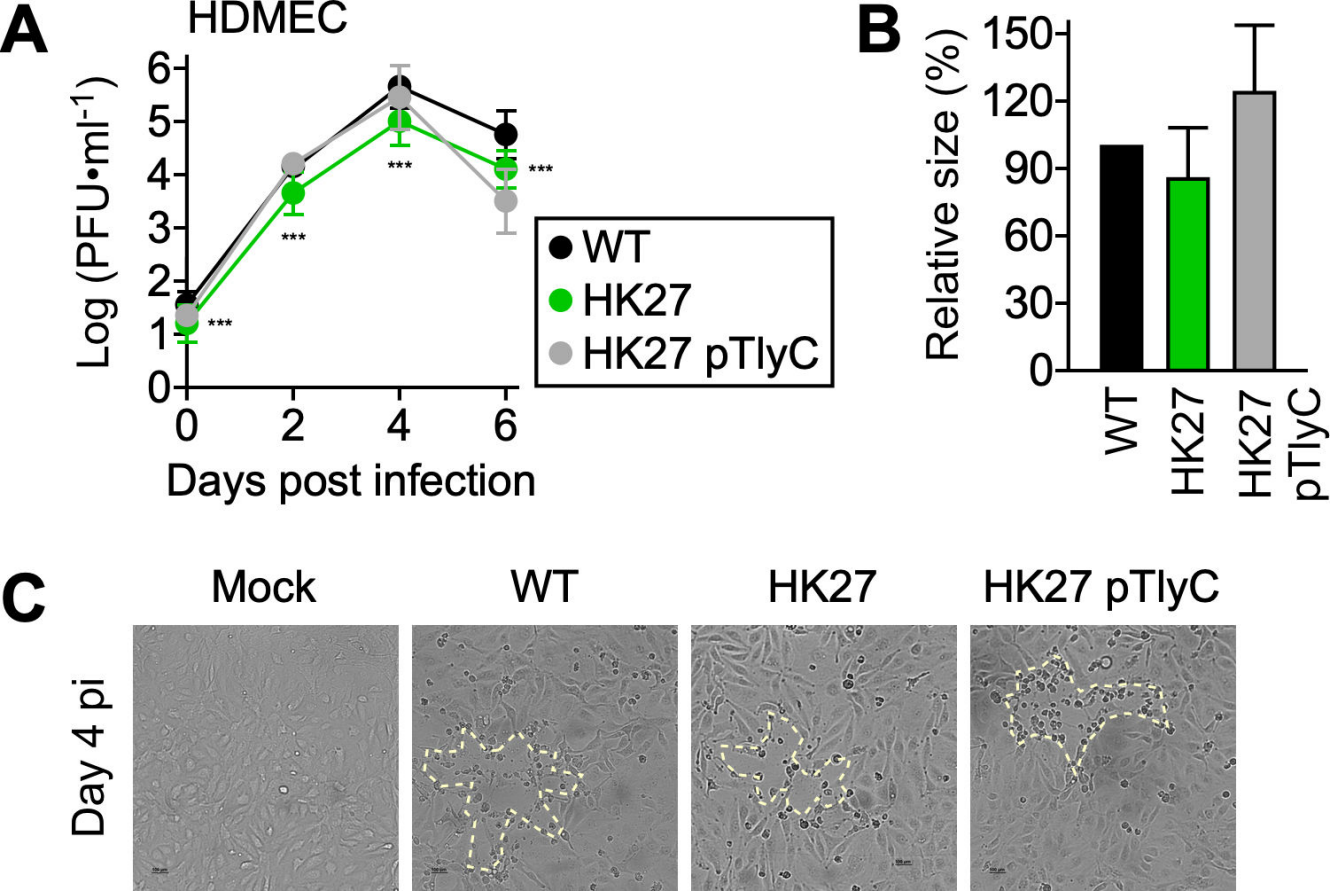
Defective intracellular replication of HK27 in endothelial cells. (**A**) Primary HDMECs (*N* = 6 [WT], 6 [HK27], and 3 [HK27 pTlyC], mean ± SD) were infected with *R. conorii* WT, HK27, or HK27 pTlyC at an MOI of 0.01 to determine rickettsial replication at timed intervals. Two-way ANOVA with Sidak’s multiple comparisons test was performed, ***P < 0.001. (**B**) Relative plaque sizes were quantified (*N* = 11 [WT], 11 [HK27], and 13 [HK27 pTlyC], mean ± SEM). One-way ANOVA with Dunnett’s multiple comparison test was performed. (**C**) Representative microscopic images of HDMECs infected with *R. conorii* WT or HK27 on day 4 post-infection. Plaques are marked with yellow dotted lines.

Here, we examined the biological roles of TlyC during intracellular infections of endothelial cells, which are the primary target cells mediating host immunity against rickettsial infections. For this, we characterized HK27 growth phenotypes by performing pairwise comparisons of bacterial replication at timed intervals in primary human dermal microvascular endothelial cells (Fig. 4A). When inoculated at an MOI of 0.01, *R. conorii* WT expanded rapidly, reaching a maximal infectious titer (5.64 ± 0.42 PFU∙mL^−1^) on day 4 post-infection. Notably, HK27 exhibited moderate levels of defective invasion and intracellular survival, producing lower infectious titers throughout the infection (*R. conorii* WT vs HK27, 4.99 ± 0.44 PFU∙mL^−1^ on day 4 post-infection, *P* < 0.001). As expected, the introduction of pTlyC into HK27 restored defective intracellular replication (*R. conorii* WT vs HK27 pTlyC, 5.45 ± 0.60 PFU∙mL^−1^ on day 4 post-infection, *P* > 0.05). Despite the significant differences in infectious titers, *R. conorii* WT, HK27, and HK27 pTlyC infections displayed comparable levels of cytopathology, leading to significant endothelial cell death and reduced plaque numbers on the sixth day of infection (Fig. 4B and C). To determine if HK27 reversion (e.g., loss of *kkaebi* transposon) contributed to cytopathology, we expanded *R. conorii* WT, HK27, and HK27 pTlyC without antibiotic treatments for 16 days. Our PCR analyses confirmed the absence of *R. conorii* WT in HK27 variants and the presence of the *kkaebi* transposon within *tlyC* in HK27 and HK27 pTlyC (Fig. S1). Our analysis also confirmed that pTlyC is maintained in HK27 pTlyC without antibiotic selection. In summary, our data suggest that TlyC contributes to rickettsial intracellular survival in endothelial cells.

### TlyC contributes to spotted fever pathogenesis

**Fig 5:**
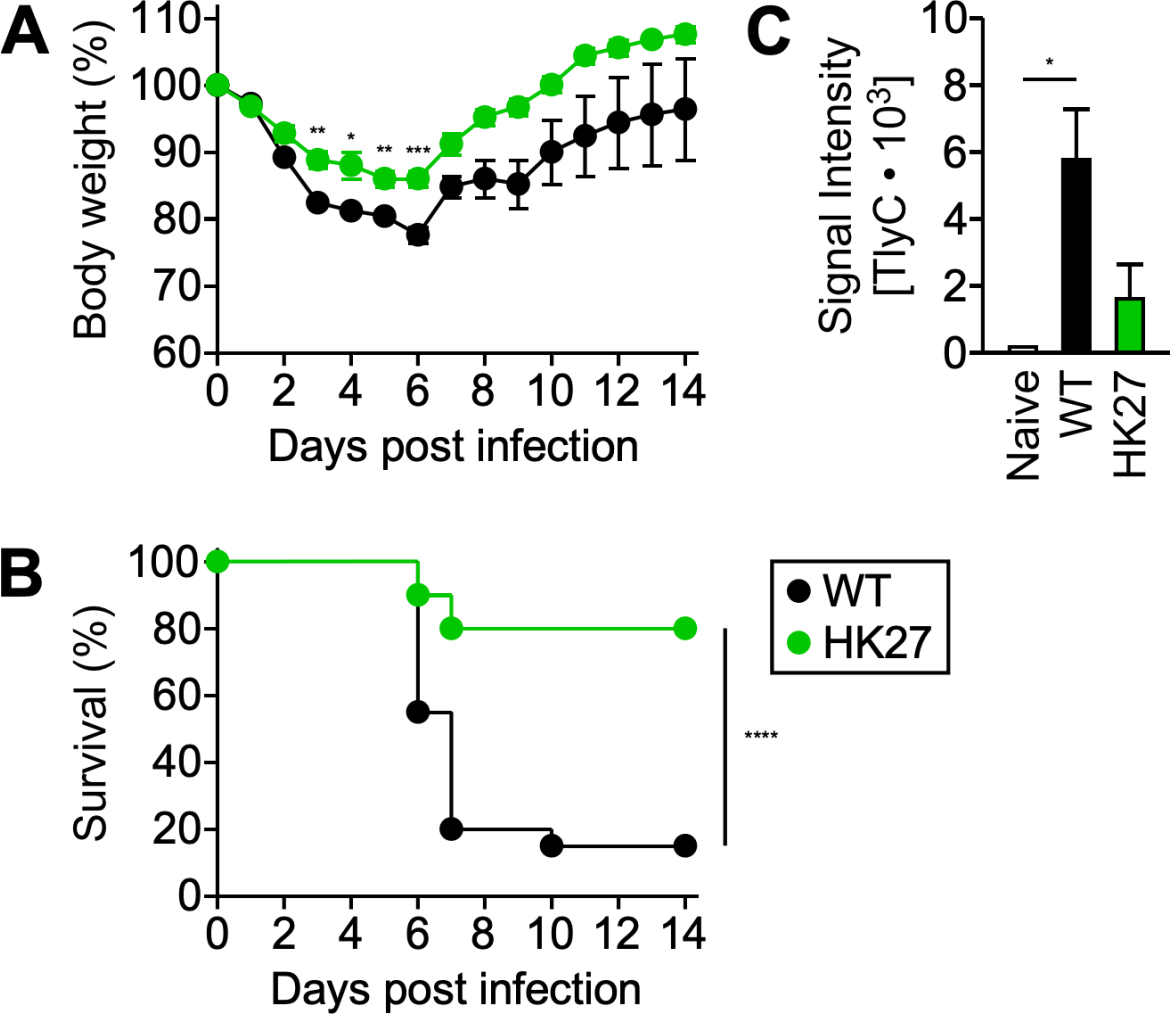
TlyC contributes to spotted fever pathogenesis in mice. Cohorts of C3H mice (*N* = 20, 10 male and 10 female) were intravenously injected with 1 × 10^3^ PFU *R. conorii* WT or HK27 and monitored for 14 days to document (**A**) body weight changes (mean ± SEM) and (**B**) mortality. (**C**) Western blot analyses determined TlyC-specific IgG levels in hyperimmune sera collected from *R. conorii*-infected (*N* = 3) or HK27-infected mice (*N* = 5) on day 14 post-infection. Two-way ANOVA with Tukey’s multiple comparisons and log-rank tests was performed. *P < 0.05, **P < 0.01, ***P < 0.001, and ****P < 0.0001.

To investigate whether HK27 exhibits virulence defects in the mouse model for acute disease, cohorts of C3H mice were intravenously inoculated with 1 × 10^3^ PFU *R. conorii* WT or HK27. Animals infected with *R. conorii* WT exhibited acute body weight loss and lethal outcomes (Fig. 5A and B). In contrast, HK27 infections resulted in moderate body weight reduction, accompanied by a significant decrease in mortality (85% mortality vs 20% mortality by day 14, *P* < 0.0001). After the acute phase, surviving animals infected with *R. conorii* WT recovered slowly over the next seven days (returning to 96% of the original body weight on day 14). On the other hand, mice infected with HK27 recovered faster, reaching their original body weight by 10 days post-infection. Of note, *R. conorii* WT infections induced TlyC-specific IgG responses, which were significantly reduced in mice infected with HK27 (Fig. 5C). Our *in vivo* mouse infection studies suggest that TlyC contributes to spotted fever pathogenesis in mice.

## DISCUSSION

PFTs have been extensively studied for their contributions to bacterial pathogenesis (16). Since the initial description of rickettsial hemolysis, a series of studies characterized biological properties affecting the hemolytic activities of TG rickettsiae (10). These studies documented that rickettsial hemolysis requires metabolically active rickettsiae and occurs in a manner dependent on bacterial concentration, rickettsial adsorption, pH, temperature, and host species, prompting the development of a rapid hemolysin test for TG *Rickettsia* (10–14). Furthermore, experimental evidence suggested that factors mediating hemolysis could not be dissociated from rickettsial bodies and displayed a modest hemolytic kinetics with incomplete hemolysis (14). In this regard, rickettsial hemolysis is different from that caused by secreted bacterial cytolysins, many of which cause rapid and complete hemolysis. With the advances in whole-genome sequencing and bioinformatic analyses, investigators identified TlyC as a putative hemolysin, predicted to assist the intracellular invasion of TG rickettsiae (17, 18). By genetic engineering, recent studies demonstrated that rickettsial phospholipases, another group of enzymes with membranolytic functions, contribute to rickettsial invasion, escape from endocytic vacuoles and autophagosomes, cell-to-cell spread, and pathogenesis (18, 21, 22). Bacterial factors capable of disrupting host membrane integrity play important roles in pathogenesis. For instance, *L. monocytogenes* secretes listeriolysin O, a PFT that disrupts the phagocytic membrane, for successful invasion into the host cytosol (23). However, the molecular mechanisms by which rickettsial hemolysins contribute to intracellular survival remain unclear.

All PFTs exhibit functional metamorphosis: (i) soluble monomers engage receptors on the target cellular membrane, (ii) monomers form multimeric pre-pore structures, and (iii) transmembrane pores form to penetrate the membrane. Although cholesterol has been implicated as a receptor for *R. prowazekii* hemolysins, the underlying molecular mechanisms associated with rickettsial hemolysin-receptor interactions, oligomerization processes, and membrane disruption remain largely unknown (15). Unlike conventional CDCs, rickettsial TlyC polypeptides do not have a conserved undecapeptide sequence (ECTGLAWEWWR) involved in cholesterol interactions and lack structural features shared among conventional CDCs in gram-positive pathogens (24, 25). Interestingly, TlyC-mediated hemolysis occurred most efficiently with human RBCs, corroborating previous observations that rickettsial hemolysins exhibit host tropism (10). Since cholesterol levels in RBCs exhibit minimal species variation, cholesterol alone cannot explain the host tropism (26). Host species-specific cytolytic activities have been documented for CDCs (for instance, intermedilysin of *S. intermedius*) interacting with human CD59, a glycosyl-phosphatidylinositol-anchored membrane protein that interacts with complement proteins C8a and C9 to inhibit the formation of the membrane attack complex (27). Thus, TlyC may interact with a specific protein, lipid, or glycan receptor to recognize the target cells and bind cholesterol to form the lytic complex on the membrane (28). In our analysis, we determined that the first 10 amino acids of TlyC are highly conserved and critical for rickettsial hemolysis. However, it remains to be determined whether the first 10 amino acids are involved in receptor recognition, oligomerization, or pore formation. Investigating TlyC receptor biology will provide a unique opportunity to understand the functional roles of human-specific rickettsial hemolysis.

In our recent study, we developed a *kkaebi* transposon insertional mutagenesis scheme using *R. conorii* as a model organism to identify rickettsial genes involved in spotted fever pathogenesis (5). In the present study, we characterized an *R. conorii* variant (HK27) with a *kkaebi* insertion in *tlyC* (*Rc1141*). Despite the transposon insertion occurring at the 3′-end of the gene (position 859 of 900 nucleotides), polyclonal rabbit antibodies raised against recombinant TlyC_Δ2-51_ failed to detect the presence of truncated TlyC variants in HK27. Similarly, immunoblot analysis failed to detect TlyC in HK27 transformed with a plasmid harboring *tlyC*_Δ249-299_. Our results suggest that HK27 is unable to synthesize TlyC, or truncated TlyC is unstable. In our hemolysis assay, despite our best efforts to optimize experimental conditions, we encountered small variations and residual spontaneous lysis, resulting in the release of hemoglobin from RBCs. Despite these technical challenges, we demonstrate that TlyC-mediated hemolytic activities peak at 34°C–37°C and neutral pH, corroborating earlier findings. In addition, TlyC-dependent hemolysis progressed slowly and failed to reach completion. Importantly, TlyC was not required for *R. conorii* adsorption to RBCs, suggesting that additional surface molecules contribute to *R. conorii* interactions with RBCs. Thus, it remains unclear if TlyC-mediated hemolytic activities require rickettsial adsorption to RBCs.

These unique biological attributes prompted us to investigate the biological roles of TlyC during intracellular replication in endothelial cells, which are the primary target cells for rickettsial pathogenesis. Growth analyses revealed that disruption of TlyC synthesis in the HK27 mutant was associated with reduced intracellular invasion and replication in primary endothelial cells. Interestingly, despite differences in intracellular growth, *R. conorii* WT and HK27 produced comparable levels of cytopathology, presumably due to other factors exhibiting redundant functions. On the other hand, when inoculated into the bloodstream, HK27 exhibited attenuated virulence in the mouse infection model of spotted fever. Since previous studies have shown that mouse RBCs are resistant to rickettsial hemolysis, our observations suggest that TlyC may play an undetermined alternative role in the rickettsial intracellular lifecycle. To better understand TlyC’s contributions to pathogenesis, future work will focus on detailed microscopic and molecular analyses at various stages of intracellular infection. We also aim to investigate whether TlyC-mediated hemolysis directly contributes to pathogenesis (for instance, through the acquisition of nutritional iron) or whether TlyC enhances rickettsial survival within innate immune cells, such as macrophages, which are crucial for host defense against rickettsial infections. Furthermore, *in vivo* studies will assess whether the loss and complementation of *tlyC* affect rickettsial survival in peripheral organs or influence host immune responses, both of which could lead to improved disease outcomes in the murine model of spotted fever. Notably, all our experiments were performed with Renografin-purified *Rickettsia* prepared from Vero cells that exhibited substantial cellular death. Although our mouse infection studies confirmed TlyC expression and antigenic properties, additional studies are necessary to identify the regulatory mechanisms and environmental cues that modulate TlyC expression throughout the rickettsial lifecycle and to elucidate its biological functions in specific stages of intracellular infection.

Five protein translocation mechanisms have been identified in *Rickettsia*: Sec translocon, twin-arginine translocon, Type I, IV, and V secretion systems (T1SS, T4SS, and T5SS, respectively) (29). Our bioinformatic analyses failed to identify twin-arginine consensus motifs, transmembrane segments, or β-barrel domains in TlyC. Furthermore, similar approaches could not find consensus sequence tags or motifs predicted to be associated with the Sec translocon, T1SS, or T4SS. The N-terminal signal peptide plays a central role in protein secretion through the Sec machinery. Thus, despite the lack of bioinformatic evidence, we characterized two TlyC variants that lack the first 10 or 20 amino acids for protein translocation. Surprisingly, the N-terminal deletional TlyC variants successfully translocated to the outer membrane, suggesting that the first twenty amino acids are dispensable for TlyC translocation. Our data suggest that TlyC may possess an atypical secretion signal, as documented for clade-specific secretion signal motifs found in *Ehrlichia*, a group of closely related bacterial organisms in the order Rickettsiales (30). Currently, we are actively pursuing the characterization of *R. conorii* variants lacking functional T1SS or T4SS, which will assist in determining TlyC secretion pathways and significantly enhance our understanding of the substrate requirements in *Rickettsia*. Furthermore, we aim to address whether TlyC is released from the outer membrane of *Rickettsia* and functions to form pores on host cellular membranes.

In conclusion, our studies demonstrate that TlyC is a conserved hemolysin in *Rickettsia*, deposited on the outer membrane, and contributes to rickettsial intracellular replication and spotted fever pathogenesis. These results provide a concrete foundation for future investigations into understanding protein secretion mechanisms in *Rickettsia*, molecular mechanisms associated with rickettsial hemolytic activity, and functional roles of TlyC in spotted fever pathogenesis.

## SUPPLEMENTAL MATERIAL

Supplemental figure legend spectrum.00303-25-s0001.docx Figure S1 legend.

Figure S1 spectrum.00303-25-s0002.tif PCR analyses confirm the presence of the *kkaebi* transposon insertion and plasmid-borne *tlyC* in HK27 variants.

Table S1 spectrum.00303-25-s0003.docx Primers used in this study.

ASM does not own the copyrights to Supplemental Material that may be linked to, or accessed through, an article. The authors have granted ASM a non-exclusive, world-wide license to publish the Supplemental Material files. Please contact the corresponding author directly for reuse.
