# Supplementary material for: TlyC, a conserved hemolysin in Rickettsia, contributes to spotted fever pathogenesis in mice
Source: Microbiol Spectr. 2025 Aug 12;13(9):e00303-25. doi: 10.1128/spectrum.00303-25 (PMC12403716; doi:10.1128/spectrum.00303-25)
Supplement: Supplemental figure legend — Figure S1 legend. [file spectrum.00303-25-s0001.docx]

**Supplemental Figure 1. PCR analyses confirm the presence of the *kkaebi* transposon insertion and plasmid-borne *tlyC* in HK27 variants.** PCR analyses (N=3) identified the presence of (left) *tlyC* in the multiple cloning sites (MCS) of pTlyC, (middle) *kkaebi* transposon, and (right) the transposon insertions in HK27 variants.
