# Supplementary figures and images for: TlyC, a conserved hemolysin in Rickettsia, contributes to spotted fever pathogenesis in mice
Source: Microbiol Spectr. 2025 Aug 12;13(9):e00303-25. doi: 10.1128/spectrum.00303-25 (PMC12403716; doi:10.1128/spectrum.00303-25)

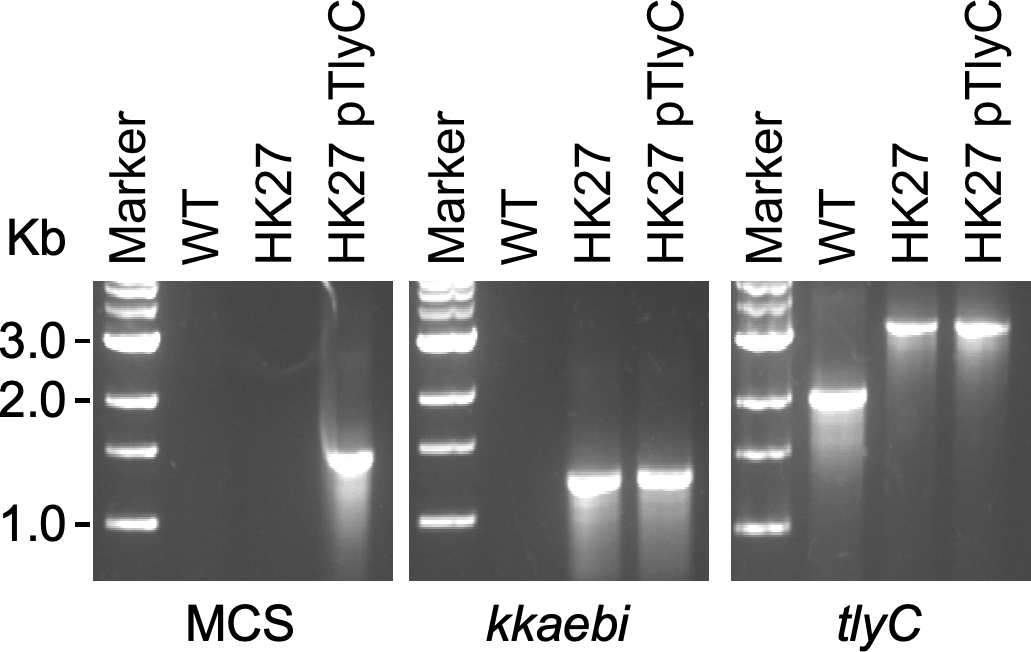

Supplement: Figure S1 — PCR analyses confirm the presence of the kkaebi transposon insertion and plasmid-borne tlyC in HK27 variants. [file spectrum.00303-25-s0002.tif]
