# Supplementary material for: TlyC, a conserved hemolysin in Rickettsia, contributes to spotted fever pathogenesis in mice
Source: Microbiol Spectr. 2025 Aug 12;13(9):e00303-25. doi: 10.1128/spectrum.00303-25 (PMC12403716; doi:10.1128/spectrum.00303-25)
Supplement: Table S1 — Primers used in this study. [file spectrum.00303-25-s0003.docx]

**Supplemental Table 1. Primers used in this study.**

| **Primer** | **Sequence (5’ – 3’)** | **Restriction Enzyme** |
| --- | --- | --- |
| TlyCF | AAACCTAGGCTAAACTTGAATTTTTAGGTGACTCTG | AvrII |
| TlyCR | AAACCTAGGCGCAAGAAAGTCCGATGATAGG | AvrII |
| pHTRL7MCSF | GGTATGGAGGTATGGATTTACCGATG |  |
| pHTRL7MCSR | CCACCTCTGACTTGAGCGTCG |  |
| 15bTIyC-DN50F | AAACATATGATTAATAGCCAAAAAATGACTTTAGACG | NdeI |
| 15bTlyCR | AAAGGATCCTCATTGACCATTCAAACCGTTTTTTAG | BamHI |
| T7Promoter | TAATACGACTCACTATAGGG |  |
| T7Terminator | GCTAGTTATTGCTCAGCGG |  |
| GenomicTlyCF | CATGACTGATCGAATAATGACAGTAAG |  |
| GenomicTlyCR | CTCAAATTTCTCTGCTAAGTCAAATGAC |  |
| Tn5F | AAAGACAGCTGTCTCTTATACACATCTCAACCATCATC |  |
| Tn5R | GACAGCTGTCTCTTATACACATCTCAACCCTGAAG |  |
